# Calnuc plays a role in dynamic distribution of Gαi but not Gβ subunits and modulates ACTH secretion in AtT-20 neuroendocrine secretory cells

**DOI:** 10.1186/1750-1326-4-15

**Published:** 2009-03-25

**Authors:** Ping Lin, Thierry Fischer, Christine Lavoie, Haining Huang, Marilyn Gist Farquhar

**Affiliations:** 1 Department of Cellular and Molecular Medicine, University of California San Diego, La Jolla, CA 92093-0651, USA; 2 Department of Immunology and Oncology, Centro Nacional de Biotecnología, Consejo Superior de Investigaciones Científicas (CSIC), Campus de Cantoblanco, 28049 Madrid, Spain; 3 Department of Pharmacology, University of Sherbrooke, Quebec, J1H 5N4, Canada; 4 La Jolla Institute for Allergy & Immunology, La Jolla, CA 92037, USA

## Abstract

In AtT-20 cells ACTH secretion is regulated by both Ca^2+ ^and G proteins. We previously demonstrated that calnuc, an EF-hand Ca^2+ ^binding protein which regulates Alzheimer's β-amyloid precursor protein (APP) biogenesis, binds both Ca^2+ ^as well as Gα subunits. Here we investigate calnuc's role in G protein-mediated regulation of ACTH secretion in AtT-20 neuroendocrine secretory cells stably overexpressing calnuc-GFP. Similar to endogenous calnuc, calnuc-GFP is mainly found in the Golgi, on the plasma membrane (PM), and associated with regulated secretion granules (RSG). By deconvolution immunofluorescence, calnuc-GFP partially colocalizes with Gαi1/2 and Gαi3 at the PM and on RSG. Cytosolic calnuc(ΔSS)-CFP with the signal sequence deleted also partially colocalizes with RSG and partially cosediments with Gαi1/2 in fractions enriched in RSG. Overexpression of calnuc-GFP specifically increases the distribution of Gαi1/2 on the PM whereas the distribution of Gβ subunits and synaptobrevin 2 (Vamp 2) is unchanged. Overexpression of calnuc-GFP or cytosolic calnuc(ΔSS)-CFP enhances ACTH secretion two-fold triggered by mastoparan or GTPγS but does not significantly affect glycosaminoglycan (GAG) chain secretion along the constitutive pathway or basal secretion of ACTH. Calnuc's facilitating effects on ACTH secretion are decreased after introducing anti-Gαi1/2, Gαi3, Gβ or calnuc IgG into permeabilized cells but not when Gα12 or preimmune IgG is introduced. The results suggest that calnuc binds to Gα subunits on the Golgi and on RSG and that overexpression of calnuc causes redistribution of Gαi subunits to the PM and RSG, indicating that calnuc plays a role in dynamic distribution of only Gα but not Gβ subunits. Thus calnuc may connect G protein signaling and calcium signaling during regulated secretion.

## Background

Calnuc (nucleobindin) [1,2], an EF-hand Ca2+ binding protein, was previously reported to bind Ca2+ and several Gα subunits *in vivo *[3,4]. Calnuc is unusual in that it is found both within the Golgi lumen and in the cytoplasm [3]. We previously demonstrated that the luminal pool of calnuc constitutes of an agonist-releasable Ca2+ store in the Golgi [5], and regulates Alzheimer's β-amyloid precursor protein (APP) biogenesis [6], whereas cytoplasmic calnuc binds several Gα subunits [3,7,8].

Transport along the regulated secretory pathway and exocytosis of secretion granules involves vesicular trafficking, fusion of secretory granules with the plasma membrane (PM), followed by release of granule contents. Regulated secretion is stimulated by Ca^2+ ^[9] and heterotrimeric G proteins, including several Gα and Gβγ subunits [10-12]. Among these, Gαi3 was found to facilitate histamine release from mast cells [13], noradrenaline release from adrenal chromaffin cells [14], and adrenocorticotropic hormone (ACTH) secretion from AtT-20 cells [15]. The recent discovery that corticotrophin releasing hormone (CRH) and vasopressin (VP) regulate ACTH secretion via binding to the Type 1 CRH receptor and the V1b receptor, which are G protein coupled receptors (GPCRs), verifies the regulation of ACTH secretion by G proteins [16]. Moreover, several G proteins have been found on intracellular membranes as well as on the PM. Gαi3 is associated with Golgi membranes as well as at the PM [17,18], and Gαi1/2 is found on secretory vesicles [14,19,20].

We have previously reported that calnuc is associated with regulated secretion granules (RSG) [21] and binds to Gαi3 in the Golgi [8]. In addition, we hypothesized that calnuc might modulate regulated secretion by virtue of its ability to bind Gαi3 and Ca^2+^. To obtain direct evidence for the role of calnuc in the regulation of G protein mediated ACTH secretion we overexpressed calnuc-GFP in AtT-20 cells. We report here that overexpressed calnuc-green fluorescent protein (GFP) partially codistributes with Gαi1/2 as well as Gαi3 on the cytoplasmic surface of regulated secretory granules (RSG), facilitates ACTH secretion triggered by the G protein activators GTPγS or mastoparan and causes redistribution of Gαi subunits by increasing Gαi1/2 on the PM and Gαi3 on RSG. Thus calnuc, the only protein demonstrated to bind both Ca^2+ ^and Gα subunits [3], appears to play an important role in regulation of G protein and Ca^2+^-related signaling events in endocrine cells.

## Results

### Distribution of Endogenous Calnuc in AtT-20 Cells and in Cells Stably Overexpressing Calnuc-GFP or Calnuc (ΔSS)-CFP

**Figure 1:**
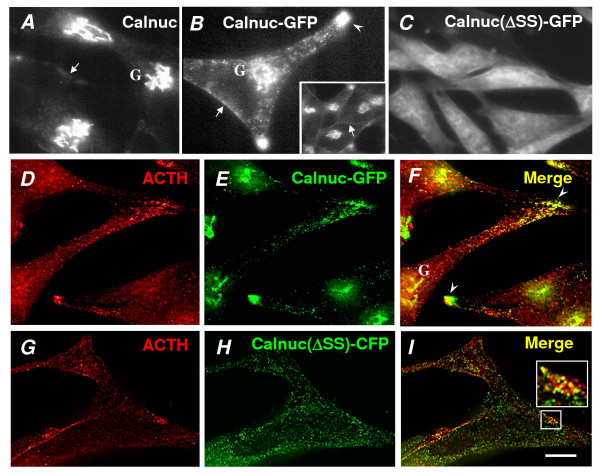
**Characterization of AtT-20 cells stably overexpressing calnuc-GFP or Calnuc(ΔSS)-CFP**. **(A)**. Immunofluorescence showing that most of the endogenous calnuc is found in the Golgi region (G) with some found on the PM (arrow) **(B) **Stably expressed calnuc-GFP in live AtT-20 cells is also concentrated in the Golgi with some found on the PM, but it is also associated with RSG stored at the tips of cell processes. **(C) **Stably expressed mutant calnuc(ΔSS)-CFP is distributed throughout the cytoplasm in live AtT-20 cells. **(D-F)**. Deconvolution analysis of immunofluorescence images demonstrates that calnuc-GFP partially colocalizes with ACTH in RSG (yellow in F) at the tips of the cell processes (arrowhead in F) and in the Golgi region (G). **(G-I)**. Deconvolution analysis of AtT-20 cells stably expressing calnuc(ΔSS)-CFP demonstrates that cytosolic calnuc is found on RSG and partially colocalizes with ACTH (yellow in I).). Bar = 10 μm.

We have previously reported [21] that most of the endogenous calnuc is concentrated in the Golgi region in AtT-20 cells (Fig. 1A) with some also associated with the PM and immature secretory granules. Calnuc-GFP directly visualized in live AtT-20 cells (Fig. 1B) or seen by deconvolution analysis of immunostained fixed sections (Fig. 1E) is similarly concentrated in the Golgi region, but it is also associated with regulated secretion granules (RSG) which contain ACTH located at the tips of the cell processes (Fig. 1D–F). In addition to its presence in the Golgi and RSG, some calnuc-GFP is also found at the PM (Fig. 1B) [8]. Our previous morphological and biochemical data established that calnuc is secreted by the constitutive-like pathway, as it is packaged into immature secretory granules in the Golgi, sorted out of RSG during their maturation, and secreted by the constitutive pathway [21].

When calnuc(ΔSS)-CFP with the signal sequence deleted which is located in the cytoplasm [8] is expressed and viewed by live cell imaging, it is seen to be distributed throughout the cytoplasm (Fig. 1C). However, when fixed cells are permeabilized before fixation (to release cytosolic calnuc), immunostained with an anti-GFP IgG, and examined by immunofluorescence and deconvolution analysis, calnuc(ΔSS)-CFP is also seen to be associated with RSG based on colocalization with ACTH (Fig. 1G–I). The findings with this mutant suggest that some of the cytosolic calnuc binds to the cytoplasmic surface of RSG. Thus calnuc appears to be located both inside RSG as well as bound to the cytoplasmic surface of RSG membranes.

### Distribution of Gαi Subunits in Parental (NT) AtT-20 Cells and Those Stably Expressing Calnuc-GFP and Calnuc(ΔSS)-CFP

**Figure 2:**
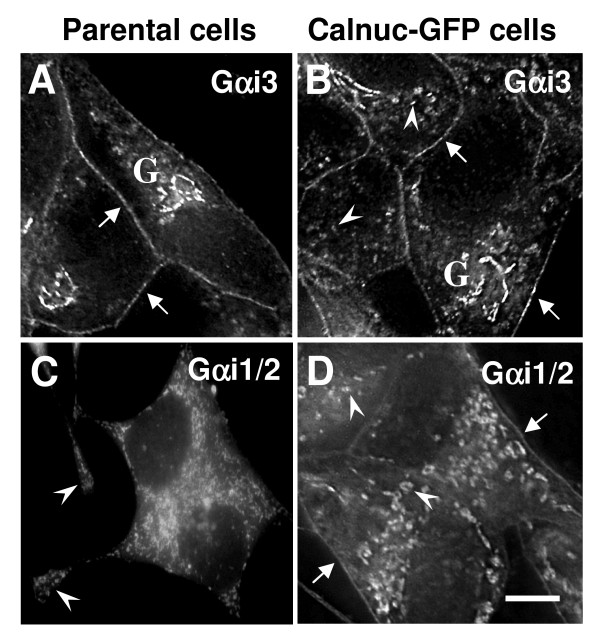
**Redistribution of Gαi subunits after overexpressing calnuc-GFP**. Gαi3 is found on the PM (arrows) and on the Golgi (G) in parental AtT-20 cells (A) and in those expressing calnuc-GFP (B). Some Gαi3 is found on RSG in cells expressing calnuc-GFP (B). Gαi1/2 is found mainly on RSG (arrows) in the cell bodies and tips of the cell processes (arrowheads) in both parental (C) and calnuc-GFP cells (D). More Gαi1/2 is observed on the PM (arrows) in cells overexpressing calnuc-GFP (D) cells than in parental AtT-20 cells (D).). Bar = 10 μm.

**Figure 3:**
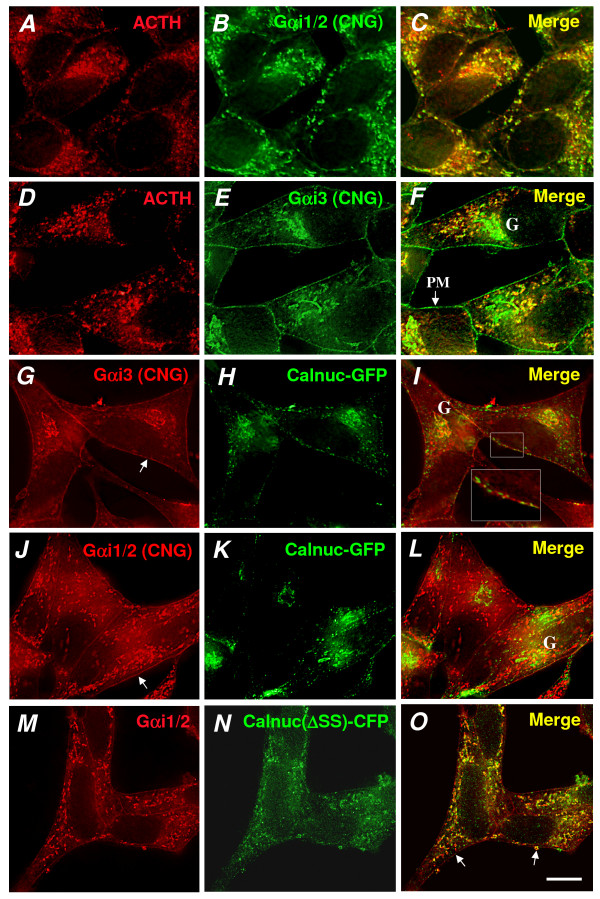
**Distribution of Gαi subunits in AtT-20 cells stably expressing calnuc-GFP or calnuc(ΔSS)-CFP**. **(A-F)**. Deconvoluting analysis of cells expressing calnuc-GFP which had been doubly stained for ACTH and endogenous Gαi1/2 or Gαi3. Both Gi subunits are associated with RSG containing ACTH (yellow in C and F). **(G-L)**. Deconvolution analysis of cells expressing calnuc-GFP and doubly stained for GFP and either Gαi3 or Gαi1/2 showing that the Gi subunits are distributed on the PM (arrows) and partially colocalize with calnuc-GFP (yellow in I and L) which is distributed in an interrupted pattern on the PM. Calnuc-GFP also colocalizes with Gαi3 on the Golgi (G). **(M-O)**. Similar deconvolution analysis of AtT-20 cells expressing calnuc(ΔSS)-CFP showing that some of the Gαi1/2 colocalizes with cytosolic targeted calnuc(ΔSS)-CFP on RSG (yellow dots in O) and the PM (arrows). Bar = 10 μm.

Next we investigated the distribution of Gα subunits in AtT-20 cells. In parental AtT-20 cells, Gαi3 is found on both the PM and the Golgi (Fig. 2A) as previously reported [21], and Gαi1/2 is associated mainly with RSG located in both the cell bodies and tips of the AtT-20 cell processes (Figs. 2C and 3A–C). In AtT-20 cells stably expressing calnuc-GFP, more Gαi3 appears to be associated with RSG (Figs. 2B and 3D–F), and both Gαi1/2 (Figs. 2D and 3A–C) and Gαi3 (Fig 2B and 3D–F) are observed on the PM as well as on RSG.

Calnuc-GFP partially colocalizes with Gαi3 in the Golgi (Fig. 3G–I) and partially co-distributes with Gαi1/2 (Fig. 3J–L) and Gαi3 (Fig. 3G–I) on RSG and the PM. In cells stably expressing cytosolic calnuc(**Δ**SS)-cyan fluorescent protein (CFP), the majority of the Gαi1/2 and membrane associated calnuc colocalize on RSG (Fig. 3M–O). Both Gαi1/2 and calnuc(**Δ**SS)-CFP are concentrated on the cytoplasmic surface of RSG and the PM. Thus, based on deconvolution analysis of our immunofluorescence results, it is evident that 1) both Gαi1/2 and Gαi3 are found on RSG where they partially colocalize with ACTH, and 2) in cells expressing calnuc-GFP the distribution of Gαi subunits along the PM and RSG is enhanced.

### Distribution of ACTH, Calnuc and Its Mutants as Well as G Protein Subunits in Membrane vs Cytosolic Fractions from Parental and Stably Transfected AtT-20 Cells

**Figure 4:**
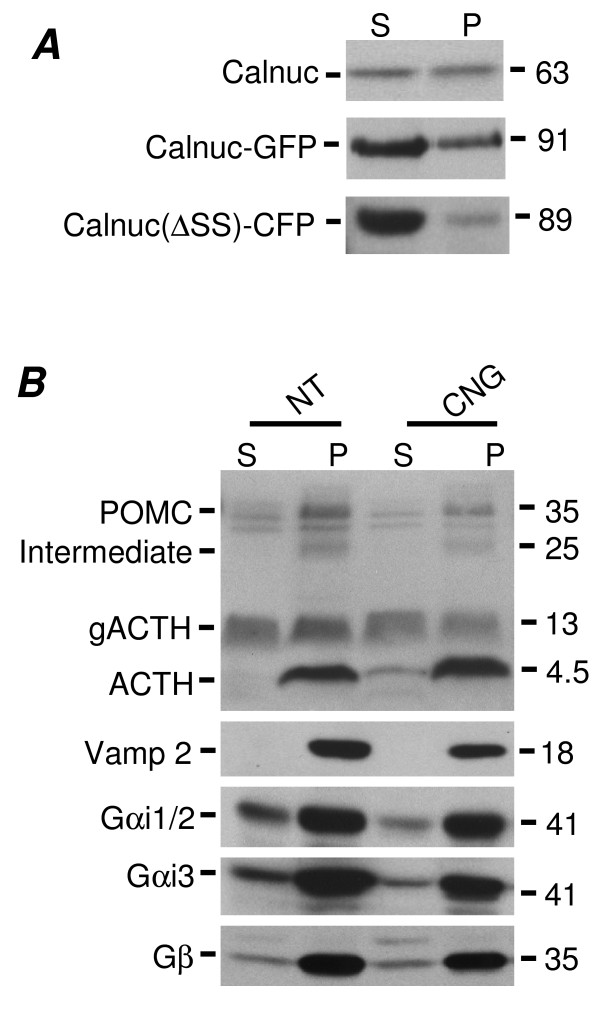
**Biochemical characterization of parental AtT-20 cells and those overexpressing Calnuc-GFP**. Postnuclear supernatants (PNS) prepared from parental AtT-20 cells or those stably overexpressing calnuc-GFP or calnuc(ΔSS)-CFP were analyzed by immunoblotting. **(A) **Endogenous calnuc (63 kD) is found in approximately equal amounts in the cytosolic (S) (100,000 × g supernatant) and membrane (P) (100,000 × g pellet) fractions. Calnuc-GFP (91 kD) is present in both fractions but is more abundant (~80%) in the cystolic fraction. Most (79%) of the mutant calnuc(ΔSS)-CFP (89 kD) is in the cytosolic (S) fraction. **(B) **The distribution of POMC, intermediate, glycosylated ACTH (gACTH) and mature ACTH is similar in parental cells (NT) cells and in cells overexpressing calnuc-GF (CNG). A small amount of ACTH is found in the cytosolic fraction of cells overexpressing calnuc-GFP, presumably due to leakage during preparation of the fractions. Synaptobrevin 2 (Vamp 2), an integral membrane protein, is found exclusively in membrane fractions. 90% of the Gβ subunits and 80% of the Gαi1/2 and Gαi3 are found in membrane fractions in parental (NT) cells. A faint band of slower mobility is seen in the case of Gβ subunits in the soluble fraction of CNG cells.

To further check the distribution and expression of calnuc and G proteins in AtT-20 cells we analyzed their distribution in membrane (100,000 × g pellet) *vs *cytosolic (100,000 × g supernatant) fractions. Figure 4A shows that endogenous calnuc (63 kD) and calnuc-GFP (91 kD) are found in both cytosolic and membrane fractions. The amount found in cytosolic fractions was greater for calnuc-GFP (80%) than endogenous calnuc (50%). Most (>90%) of the calnuc(**Δ**SS)-CFP (89 kD) was found in the cytosolic fraction, but ~10% pelleted with membranes in keeping with the immunofluorescence results demonstrating its association with RSG. The distribution of proopiomelanocortin (POMC)-derived products including POMC, POMC intermediate, glycosylated ACTH (gACTH) and mature ACTH was also checked and was similar in parental AtT-20 cells and those overexpressing calnuc-GFP (Fig. 4B). Synaptobrevin 2 (Vamp 2) which localizes on both RSG and synaptic vesicles was found exclusively in membrane fractions (Fig. 4B). 80% of the Gαi1/2, Gαi3 and Gβ subunits were associated with membrane fractions in parental AtT-20 cells and 90% in calnuc-GFP cells (CNG). An additional Gβ band was also found in the calnuc-GFP cells. We conclude that overexpression of calnuc-GFP does not significantly change the expression level or localization ratio (membrane vs cytosolic fractions) of Gαi subunits, ACTH or its derivatives.

### Overexpression of Calnuc-GFP Does Not Affect Constitutive Secretion of GAG Chains or Basal Secretion of gACTH and Its Precursors in AtT-20 Cells

**Figure 5:**
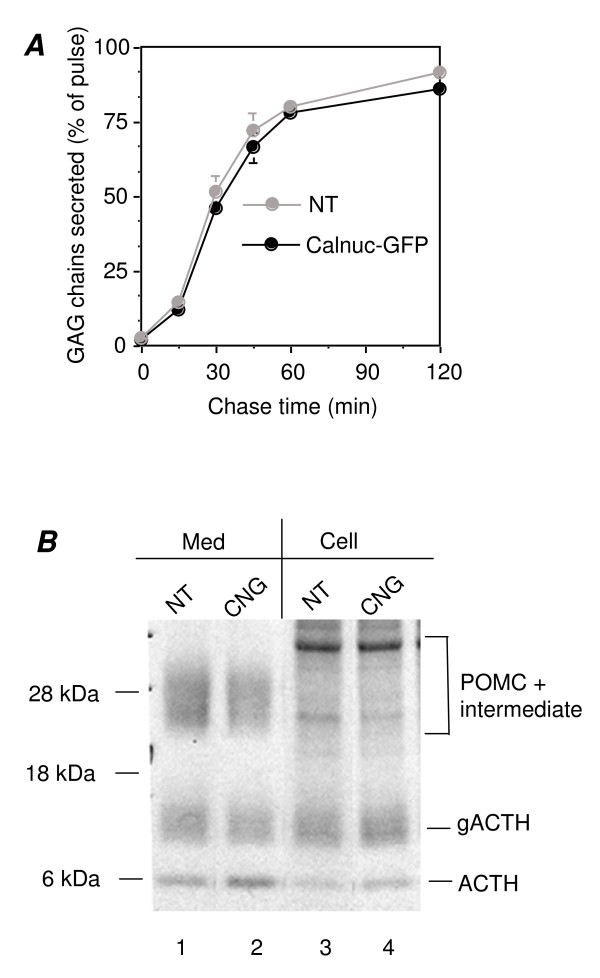
**Calnuc does not affect constitutive secretion of GAG chains or basal secretion of gACTH and its precursors**. **(A) **Overexpressed calnuc does not affect constitutive secretion of glycosaminoglycan (GAG) chains from either parental AtT-20 cells (NT) or those stably transfected with calnuc-GFP over the time period indicated (15 min to 2 h). ~85% of the GAG chains were secreted from both cell types at 2 h. Results (mean ± SD) represent the average of values obtained in 3 separate experiments. Cells were pretreated with 0.5 mM xyloside and subsequently pulse-labeled with [^35^S]sulfate for 5 min. Secreted GAG chains were precipitated with cetylpyridinium chloride. Samples collected by vacuum filtration were counted by liquid scintillation. **(B) **Comparing to parental cells, in those overexpressing calnuc, no significant difference of gACTH and POMC + intermediate is observed both extracellularly (Lanes 1 and 2) and intracellularly (Lnaes 3 and 4). However, relatively increased secreted (Lane 2) and intracellular (Lane 4) mature ACTH are shown when calnuc is overexpressed. Med: medium; Cell: intracellular.

Next we examined the effects of overexpression of calnuc-GFP on secretion of glycosaminoglycan (GAG) chains, commonly used as a marker of constitutive secretion from AtT-20 cells [22] and on basal secretion of ACTH and its precursors. No change was detected in GAG secretion, as similar amounts of GAG chains were secreted from both parental AtT-20 cells and those expressing calnuc-GFP over a period of 15 min to 2 h (Fig. 5A). The intracellular and basal secreted metabolically labeled POMC, intermediate, glycosylated ACTH (gACTH) was also similar in parental (NT) and in calnuc-GFP (CNG) cells after a 2 h chase. However, certain increased secreted (Lane 2) and intracellular mature ACTH (Lane 4) were observed following overexpression of calnuc (Fig. 5B).

### Overexpression of Calnuc-GFP Increases ACTH Secretion from AtT-20 Cells Stimulated by Mastoparan or GTPγS and the Effect Is G protein-dependent

**Figure 6:**
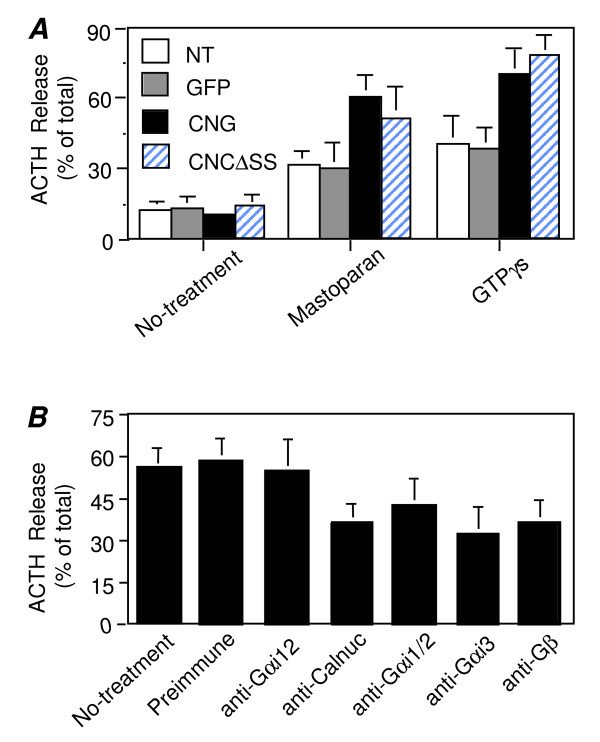
**Calnuc-GFP or Calnuc(ΔSS)-CFP increases ACTH secretion triggered by mastoparan or GTPγS and the effect is G protein-dependent**. **(A) **The amount of total ACTH secreted after stimulation with mastoparan increases from 30% in parental (NT) cells or those expressing GFP alone to 60% and 50% in AtT-20 cells overexpressing calnuc-GFP and calnuc(ΔSS)-CFP (CNGΔSS), respetively. Similar effects are observed after stimulation with GTPγS where 70–75% of the total ACTH is secreted in cells overexpressing calnuc-GFP (CNG) or calnuc(ΔSS)-CFP cells *vs *40% in parental cells (NT) or those expressing GFP alone. Results (mean ± SD) represent the average of values obtained in 3 separate experiments performed in triplicate. **(B)**. Introduction of anti-calnuc or anti-G protein antibodies decreases ACTH secretion triggered by mastoparan. In control cells (no-treatment) or those treated with preimmune or anti-Gα12 IgG, 55–60% of the total ACTH was secreted. After pre-treatment with anti-calnuc, anti-Gαi1/2, anti-Gαi3 and anti-GβIgG, ACTH secretion triggered by mastoparan was decreased to 37%, 43%, 32% and 37%, respectively. Results (mean ± SD) represent the average of values obtained in two separate experiments performed in duplicate.

To investigate if calnuc plays a role in regulated secretion we used permeabilized, parental AtT-20 cells and those overexpressing calnuc-GFP or calnuc(**Δ**SS)-CFP. Cells were permeabilized with digitonin, and ACTH secretion was triggered with the wasp venom peptide mastoparan which mimics receptor activation of G proteins by binding to the C-terminus of Gαi and Gαq [23]. Mastoparan has been used to study exocytosis in AtT-20 by others [15]. We found that when cells were stimulated with mastoparan, 30% of the total ACTH was secreted by parental cells whereas the amount secreted by cells expressing calnuc-GFP was ~60% of the total or double that found in parental cells (Fig. 6A). The amount secreted by cells expressing calnuc(**Δ**SS)-CFP was also increased (~55% of total). ACTH secretion was similarly increased in calnuc-GFP or calnuc(ΔSS)-CFP cells after stimulation with GTPγS (another G protein activator), where 70–75% of the total ACTH was secreted *vs *40% in parental cells or those overexpressing GFP alone (Fig. 6A). The result indicates that calnuc enhances regulated secretion of ACTH triggered by the G protein activators mastoparan and GTPγS.

Next we investigated whether the effects of calnuc on ACTH secretion is G protein dependent by introducing affinity purified antibodies against Gαi, Gβ or calnuc into the cytoplasm of calnuc-GFP cells. ACTH secretion triggered by mastoparan from cells pre-treated with anti-calnuc, anti-Gα1/2, anti-Gαi3 and anti-GβIgG decreased by 42%, 33%, 50%, and 42% respectively, compared to non-treated AtT-20 cells or those treated with preimmune IgG (Fig. 6B). No significant change in ACTH secretion was seen from non-stimulated calnuc-GFP cells or those pre-treated with either anti-Gα12 as a negative control or calnuc preimmune IgG. Together the results obtained with mastoparan treatment and antibody inhibition support the conclusion that calnuc as well as G protein subunits stimulate regulated secretion of ACTH in AtT-20 cells.

### Calnuc Does Not Regulate G protein Activity

**Figure 7:**
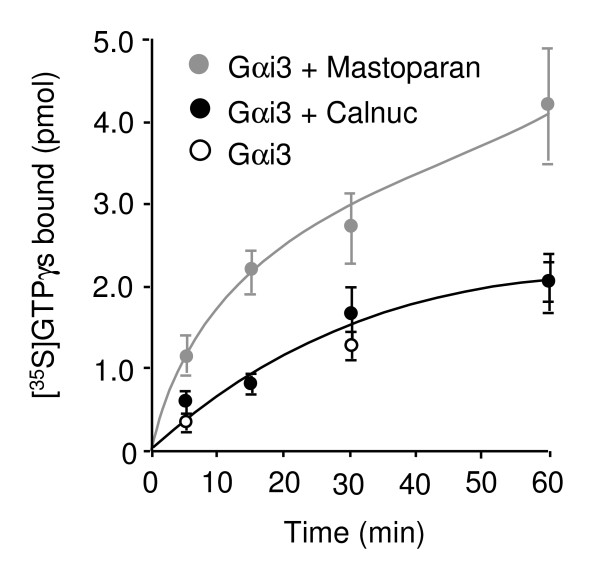
**Calnuc does not affect binding of GTPγS to Gαi3**. Addition of calnuc to Gαi3 has no effect on its rate of GDP exchange and binding of GTPγS, whereas mastoparan (used as a positive control) greatly enhances binding of GTPγS to Gαi3. Results (means ± SD) represent the average of values obtained in 3 separate experiments after subtraction of the blank (0.1% of the total radioactivity added). Mastoparan (100 μM) or His6-calnuc (1 μM) was incubated with 2 μM [^35^S]GTPγS. Reactions were started by adding 200 nM His6-Gαi3 and incubated at 30°C for 0–60 min. 50 μl of the reaction mixture were collected on nitrocellulose filters by vacuum filtration. Bound radioactivity was determined by liquid scintillation counting.

We next investigated if calnuc's effects on ACTH secretion triggered by mastoparan and GTPγS are due to regulation of G protein activity. Calnuc was previously demonstrated by us to bind to the same region of the C-terminal α5-helix on Gαi3 as mastoparan [7]. We therefore investigated if purified, recombinant calnuc behaves similarly to mastoparan (Fig. 7). Mastoparan was found to increase GTPγS binding to Gαi3 over a 5 to 60 min period. 5 min after the reaction was started, mastoparan increased binding of GTPγS to Gαi3 4.15 ± 1.33 fold, which is similar to that described for Gαi1 [23]. However, in contrast to mastoparan or AGS3 (Activator of G protein signaling 3) [24], no effect was observed when calnuc was added in the same assay (Fig 7). This result indicates that although it binds Gαi *in vivo *[8], it most likely does not regulate G protein activity. In addition, calnuc was not found to possess GAP activity (data not shown). We conclude that although calnuc binds Gα subunits, it most likely does not act as a GDI, GEF or GAP for these G proteins.

### Calnuc-GFP Localizes in Both Light and Heavy Membrane Fractions

**Figure 8:**
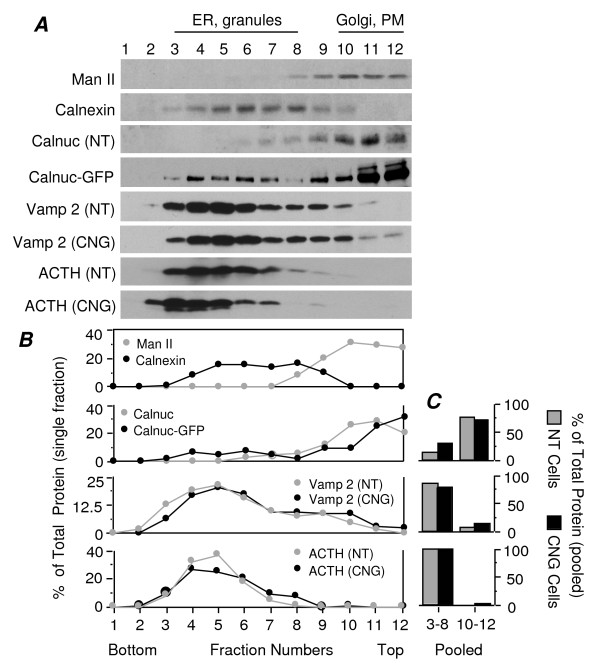
**Overexpressed calnuc-GFP is concentrated in light membrane fractions containing plasma membranes and Golgi membranes**. **(A) **Majority (90%) of the endogenous calnuc (NT) is in light fractions 10–12 containing Golgi and PM, with the remaining 10% found in heavy fractions (3–8). Calnuc-GFP is similarly concentrated in light fractions (75%), but some (25%) is also found in heavy fractions 3–8 containing ER and RSG, where it cosediments with synaptobrevin 2 (Vamp 2) and ACTH, a marker for RSG. No major change in the distribution of Vamp 2 and ACTH is observed following overexpression of calnuc-GFP. The ER marker calnexin and the Golgi marker α-mannosidase II (Man II) are found in heavy fractions (3–8) and light fractions (9–12), respectively. Overexpression of calnuc-GFP was not found to significantly change the distribution of ACTH and Vamp in the gradients. The percent of the total for a given protein in each quantified fraction was plotted in **(B)**. The amount of each protein found in fractions 3–8 and 10–12 were pooled and plotted in the bar graph as the percent of total as shown in **(C)**. Similar distribution change of indicated proteins was observed in 3 separate experiments depicted.

Our findings that calnuc increases ACTH secretion and anti-G protein antibodies partially block this effect, suggests that calnuc's effects on ACTH secretion occur via G proteins. Because our immunofluorescence results (Fig. 2) suggested a shift in the distribution of Gα subunits occurs following overexpression of calnuc-GFP, we analyzed the effects of overexpressing calnuc on G protein distribution by subcellular fractionation. Initially we used a sucrose gradient centrifugation protocol [25] aimed at separating Golgi and PM (light fractions) from ER and RSG (heavy fractions). In keeping with the immunofluorescence results, we found that the majority of the endogenous calnuc (>90%) found in parental AtT-20 (NT) cells is concentrated in light fractions (9–12) containing Golgi and PM (Fig. 8) where it cosediments with the Golgi marker α-mannosidase II (Man II). Overexpressed calnuc-GFP is also concentrated mostly in light fractions (>90%), but some (<10%) is also found in heavy fractions (3–8) containing ER and granules where it cosediments with calnexin, an ER marker, and ACTH and synaptobrevin 2 (Vamp 2), markers for RSG. This is in keeping with our finding that calnuc-GFP colocalizes with RSG. The distribution in the gradient and level of expression of Vamp 2 and ACTH are not significantly changed following overexpression of calnuc-GFP. The results indicate that unlike endogenous calnuc mainly localize in the Golgi, overexpressed calnuc localzed in both heavy (ER and granules) and light (plasma membrane and Golgi) fraction. However, overexpression of calnuc doesn't change ACTH and Vamp 2 distribution.

### Overexpression of Calnuc Affects Distribution of Gαi Subunits

**Figure 9:**
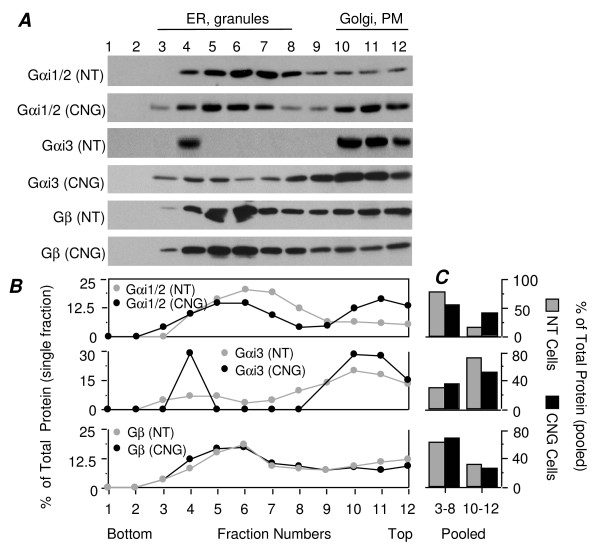
**Redistribution of Gαi subunits in AtT-20 cells overexpressing calnuc-GFP**. In parental AtT-20 cells (NT), the majority of the endogenous Gαi1/2 (70%) is found in heavy fractions (3–8) containing ER and secretory granules, whereas most of the endogenous Gαi3 (72%) is present in light fractions (10–12) containing Golgi and PM. In cells overexpressing calnuc-GFP (CNG) the % of the total Gαi1/2 found in heavy fractions is reduced (55%), that in light fractions is correspondingly increased (41%), and Gαi3 is more broadly distributed in fractions 3–12. No obvious change in the distribution of Gβ is observed following calnuc-GFP overexpression. Similar distribution change of indicated proteins was observed in 3 separate experiments depicted. The quantitative analysis shown by bar graph which was calculate based on the dot curve in left panel represent the result from the same illustrated experiment as indicated above. The experimental protocol is the same as for Figure 6. "B" and "C" were calculated and plotted as in Figure 8A.

We next examined whether the distribution of G proteins changes upon overexpression of calnuc-GFP using the same sucrose gradient protocol. Figure 9 shows that in parental AtT-20 cells (NT), the majority of the Gαi1/2 (70%) is in heavy fractions (3–8) containing ER and RSG, and the majority of the endogenous Gαi3 (72%) sediments in the light fractions (10–12) containing Golgi and PM. In cells overexpressing calnuc-GFP, the total Gαi1/2 in the heavy fractions is reduced to 55% with a corresponding increase in light fractions to 41%. In addition, Gαi3 is found to have a broader distribution (fractions 3–12) in heavier fractions in calnuc-GFP cells, which is consistent with the localization of Gαi3 on RSG in these cells (see Figs. 3D–F). Gβ subunits are broadly distributed throughout the gradient in both heavy and light fractions, and their distribution is unchanged following overexpression of calnuc-GFP. These findings indicate that overexpression of calnuc-GFP results in increased Gαi1/2 found in light fractions containing Golgi and PM and a broader distribution of Gαi3 in heavy fractions.

### Overexpression of Calnuc-GFP Increases Distribution of Gαi1/2 on the PM in AtT-20 Cells

**Figure 10:**
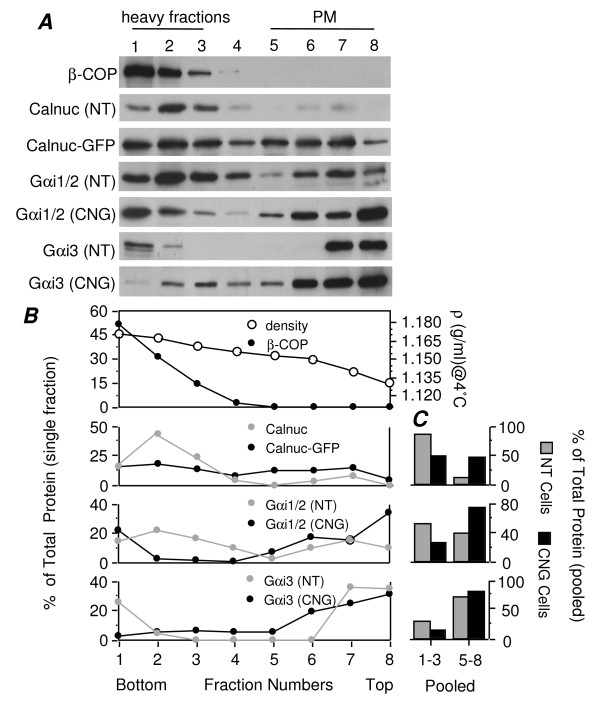
**Total Gαi1/2 associated with PM is increased following overexpression of calnuc-GFP**. Light fractions enriched in PM were separated from heavy fractions containing Golgi and ER by sucrose gradient flotation. In parental cells (NT), the Golgi marker (β-COP) and most (84%) of the endogenous calnuc are concentrated in heavy fractions. In cells stably overexpressing calnuc-GFP (CNG), Calnuc-GFP is distributed across both light and heavy fractions, and the % of the total Gαi1/2 in PM-enriched fractions (5–8) is increased from 38% to 63%. Most of the Gαi3 (75%) is found in PM fractions with the remaining (25%) associated with the Golgi in parental cells. Gαi3 has a broader distribution (fractions 1–8) in calnuc-GFP cells (CNG). Postnuclear supernatants in 1.3 M sucrose were overlayed with 1.2 M, 1.14 M, 0.99 M and 0.9 M sucrose, followed by centrifugation at 170,000 × g for 15 h. Eight fractions were collected by centrifugation (100,000 × g for 1 h), and membrane pellets were analyzed by SDS-PAGE followed by immunoblotting. "B" and "C" were plotted as in Figure 6.

To further distinguish whether the increased Gαi1/2 found in light fractions is associated with Golgi membranes or the PM, we used a sucrose gradient flotation method [26] designed to separate light fractions (5–8) enriched in PM from heavy fractions (1–3) containing Golgi and ER. The % of the total Gαi1/2 in PM fractions (fractions 5–8) increased from 38% to 63% following overexpression of calnuc-GFP (Fig. 10). No striking change in the distribution of Gαi3 in fractions 6–8 (95% in calnuc-GFP cells vs. 90% in parental cells) was observed except that a broader spread throughout the gradient (fractions 1–8) was seen, similar to that of calnuc-GFP. In parental cells, the Golgi marker (β-COP) and most of the endogenous calnuc (84%) was concentrated in heavy fractions in keeping with its Golgi localization. These results together with the immunofluorescence data (Figs. 2C–D) support the conclusion that overexpression of calnuc leads to a shift in the distribution of Gαi1/2 from RSG to the PM.

## Discussion

Calnuc, the first protein identified that binds to both Ca^2+ ^and heterotrimeric G proteins, was previously shown to be localized both in the Golgi and in the cytoplasm [3]. Our previous studies established that cytoplasmic calnuc specifically interacts with several Gα subunits *in vivo *as shown by both co-immunoprecipitation [3,4] and FRET analysis [8] and that calnuc binds the C-terminal α5-helix region of Gαi3 through its EF-hand Ca^2+^-binding region [7].

In this study we focused on defining the role of calnuc in G protein dependent activation of ACTH secretion and used GTPγS or mastoparan [15,23], a receptor mimetic activator of Gi/o subunits, to trigger ACTH secretion. We found that overexpression of calnuc increased nearly two-fold ACTH secretion induced by mastoparan or GTPγS compared to non-transfected cells. The fact that no differences were seen between calnuc-GFP (located both within organelles along the secretory pathway and in the cytoplasm) and calnuc lacking its signal sequence (located exclusively in the cytoplasm) together with our finding that ACTH secretion is reduced when anti-calnuc antibodies are introduced into permeabilized cells demonstrate that it is the cytosolic pool that is responsible for calnuc's effects on secretion. By immunofluorescence we found that some of the calnuc(ΔSS)-CFP is associated with the cytoplasmic side of RSG, indicating that this association must occur by protein-protein interaction between calnuc and a binding protein found on the cytoplasmic surface of the RSG membrane.

Using antibodies against Gαi2 or Gαi3 we were able to similarly impair ACTH secretion, but antibodies against Gαi12 were without effect because mastoparan does not activate Gα12. We found that mastoparan increases the initial rate of GTPγS binding to Gαi3 four-fold compared to Gαi3 alone or in the presence of calnuc. Mastoparan does so by unwinding the α5 helix of Gαi1 which is highly homologous to Gαi3 [23]. Although mastoparan and calnuc have the same binding site in the α5 helix of Gαi3, it appears that mastoparan and calnuc have different mechanisms regarding regulation of ACTH secretion because unlike mastoparan, calnuc can not regulate G protein activity but affects G protein distribution.

We also investigated the effects of overexpression of calnuc-GFP on G protein distribution. Our immunofluorescence results demonstrate that in AtT-20 cells stably expressing calnuc-GFP, calnuc-GFP partially colocalizes with Gαi1/2 as well as Gαi3 on the PM and Golgi membranes which is similar to what we reported earlier in EcR-CHO cells overexpressing calnuc and Gαi3-GFP [7] or in COS-7 cells overexpressing calnuc-GFP and Gαi3-YFP visualized by FRET [8]. Here we show (see Fig. 2C) that in non-transfected AtT-20 cells Gαi1/2 is found mainly on secretory granules (RSG) but not the PM which is consistent with reports by others on chromaffin cells [14,19] and rat melanotrophs [20]. However, in AtT-20 cells overexpressing calnuc the distribution of Gαi1/2 is shifted in that more is found on the PM and on RSG based on results obtained by both immunofluorescence (Fig. 2D) and cell fractionation (Figs. 9, 10). When calnucΔSS-GFP lacking the calnuc signal sequence is overexpressed in the cytoplasm, the majority of Gαi1/2 was found to colocalize with cytosolic calnuc on RSG. These results suggest that there is a dynamic distribution of Gαi between the cytoplasm, the PM, and membranes of subcellular compartments such as the Golgi.

**Figure 11:**
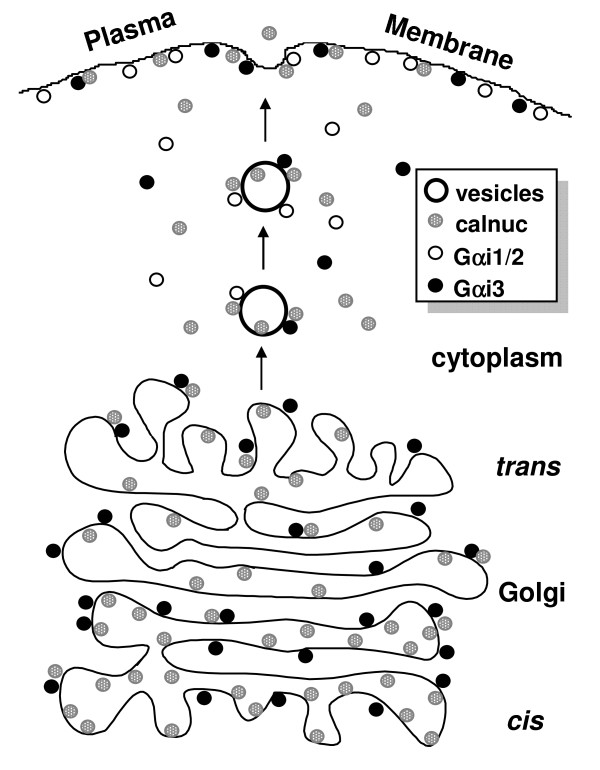
**Diagram of dynamic distribution of calnuc**. There are two pools for caluc: cytosolic and the Golgi membrane associated. Cytosolic calnuc interacts with Gαi subunits on the Golgi and vesicles budded from the Golgi, whereas luminal calnuc retains in the Golgi lumen for several hours, followed by secretion along the constitutive-like pathway. Calnuc binding the Golgi lumen is subjected to post-translational modification, and as the major calcium binding protein, plays a key role in constitution of a calcium pool in the Golgi. Cytosolic calnuc involves distribution of Gαi subunits via binding to them, and further participates regulation of G protein related intracellular pathways.

Based on our results we propose that cytosplasmic calnuc may play a role in recruiting Gαi3 onto the granules and Gαi1/2 onto the PM either from its cytosolic pool and/or from its Golgi pool (Fig. 11). However, it is possible that increasing the Ca^2+ ^concentration in the Golgi by calnuc overexpression [5] may also contribute to regulation of membrane trafficking [25,27-29] which further results in redistribution of G proteins.

Calnuc resembles several other proteins that are also localized in two pools [30,31]. We established that calnuc is distributed both in the Golgi lumen and in the cytoplasm in numerous cell lines [3,7]. Recently, NEFA [32,33] a protein that has high homology to calnuc and also has a signal sequence, has also been found both within the Golgi lumen and in the cytoplasm [34]. Several mechanisms have been proposed to explain the dual localization of the same protein [30,31,35,36]. It has been suggested that under some conditions the signal sequence may be masked by binding proteins other than SRP [35,36]. The detailed mechanism for generating two pools of calnuc remains to be elucidated.

Regulated exocytosis has been extensively investigated to date in AtT-20 and other cells [37-40]. It has become clear that it requires multiple steps that need to be controlled in time and space. Ca^2+ ^and heterotrimeric G proteins have been shown to affect exocytosis either separately or in synergy depending on the cell type. In AtT-20 cells exocytosis is dependent on both Ca^2+ ^and heterotrimeric G proteins [12,41]. We found that calnuc binds both Ca^2+ ^and heterotrimeric G proteins and is found on secretory vesicles which places it in a strategic location to serve as a vesicle bound controller of regulated secretion.

In summary, in this study we demonstrate that overexpression of calnuc, a Ca^2+ ^and heterotrimeric G protein binding protein, results in redistribution of both Gαi1/2 on the PM and Gαi3 on RSG, indicating that calnuc plays a role in dynamic distribution of only Gα but not Gβ subunits. Calnuc, which binds to Gα subunits on the vesicles, modulates G protein activator triggered ACTH secretion by redistributing Gαi1/2 and Gαi3.

## Methods

### Materials

Polyclonal rabbit IgG (F-5059) against recombinant full length calnuc was generated and affinity purified as previously described [3]. Affinity purified rabbit anti-calnuc IgG raised against the C-terminal 14 amino acids of rat/mouse calnuc (EQPPVLPQLDSQHL) or human calnuc (LLERLPEVEVPQHL) was obtained from AVIVA System Biology Corp. (San Diego, CA). Polyclonal antibodies against ACTH (UV16) and monoclonal antibody (mAb) against synaptobrevin 2 (Vamp 2) (69.1) were provided by Drs. J.D. Castle (University of Virginia, Charlottesville, VA) and P. DeCamilli (Yale University, New Haven, CT), respectively. Affinity purified IgG against Gαi1/2 (AS) and Gαi3 (EC) were gifts from Drs. Teresa Jones and A. Spiegel (NIDDK). Polyclonal anti-Gβ antibody (T-20) (Santa Cruz) which recognizes all Gβ subunits and CFP cDNA were provided by Drs. P. Insel and R. Tsien (University of California, San Diego), respectively. MAbs against ACTH and GFP were purchased from Novacastra Laboratories (Burlingame, CA) and Clontech Laboratories (Palo Alto, CA), respectively. Highly cross-adsorbed Alexa Fluor^® ^488 or 594-conjugated F(ab')_2 _fragments of goat anti-mouse or goat anti-rabbit IgG (H+L) were from Molecular Probes (Eugene, OR). Affinity purified goat anti-mouse and goat anti-rabbit IgG (H+L) conjugated to horseradish peroxidase were from Bio-Rad (Hercules, CA). Supersignal chemiluminescent reagent was purchased from Pierce (Rockford, IL). All chemicals were obtained from Sigma except as indicated.

### Cell Culture

AtT-20/D-16v pituitary cells were cultured in DME medium (high glucose) supplemented with 10% (v/v) horse serum, 2.5% (v/v) FCS (Life Technologies, Gaithersburg, MD), 100 U/ml penicillin G, and 100 μg/ml streptomycin sulfate. Cells were used as 80% confluent monolayers for transfection and subsequently selected and maintained in the same culture medium containing 0.25 mg/ml G418 sulfate (Calbiochem, La Jolla, CA).

### Establishment of AtT-20 Cells Stably Overexpressing Calnuc-GFP and Calnuc(ΔSS)-CFP

AtT-20 cells were transfected with GFP, calnuc-GFP or calnuc(ΔSS)-CFP (lacking a signal sequence) cDNAs cloned in the pcDNA3 vector, followed by G418 selection (0.75 mg/ml) for 2–3 wk as previously described [5]. Cells were subsequently sorted by FACS (Ex/Em: 488/530 ± 15) (FACSVantage SE, Beckton Dickson, San Jose, CA) in the Flow Cytometry Core Facility, UCSD Cancer Center. The highest expressors (0.12% of the positive cells) were collected and maintained in media containing 0.25 mg/ml G418. Selection by FACS sorting was repeated 3 times until 100% of the cells were positive for calnuc-GFP or calnuc(ΔSS)-CFP.

### Immunofluorescence and deconvloting analysis

Fluorescence images were collected from live AtT-20 cells stably overexpressing calnuc-GFP, calnuc(ΔSS)-CFP or GFP alone using a Zeiss Axiophot equipped with a FITC-filter (Ex/Em: 485/510).

For immunofluorescence, cells were fixed in 2% paraformaldehyde in phosphate buffer and permeabilized as previously described [3]. They were then incubated with 0.1 μg polyclonal rabbit anti-calnuc, anti-ACTH, anti-Gαi1/2(AS), and anti-Gαi3(EC), or anti-GFP mAb at room temperature for 1 h, followed by incubation with Alexa Fluor 488 or 594-conjugated anti-rabbit or anti-mouse F(ab')_2_. Specimens were examined with a Zeiss Axiophot microscope equipped for epifluorescence.

For deconvolution analysis of immunofluorescence results, images were collected with an Applied Precision DeltaVision imaging system (Issaquah, WA) coupled to a Zeiss S100 fluorescence microscope (Carl Zeiss; Thornwood, NY). Cross-sectional images of cells were obtained with 150-nm step width to optimize reconstruction of the center plane image. Deconvolution was done on a Silicon Graphics Octane^® ^visual workstation (SGI, Mountain View, CA) equipped with Delta Vision reconstruction software.

### Assessment of Constitutive Secretion of Glycosaminoglycans (GAG) from AtT-20 Cells

Non-transfected AtT-20 cells or those transfected with calnuc-GFP were pretreated with 0.5 mM xyloside at 37°C for 30 min and subsequently pulse-labeled with [^35^S] sulfate (150 μCi/ml) (ICN Biomedicals) for 5 min as described [22]. Labeled GAG chains secreted into the medium at selected intervals from 15 min to 2 h were precipitated with cetylpyridinium chloride (CPC). Samples were collected by vacuum filtration and counted by liquid scintillation as previously described [22].

### Immunoprecipitation of Metabolically Labeled ACTH and its Precursors

Parental AtT-20 cells or cells stably expressing calnuc-GFP were pulse-labeled [42] with [^35^S]Met (0.5 mCi/ml) (NEN^® ^Life Science Products, Boston, MA) for 20 min at 37°C and subsequently chased in unlabeled DMEM for 2 h. ACTH released into the medium was immunoprecipitated with anti-ACTH IgG and protein A beads (Cytelligen Corp., San Diego, CA), followed by separation on 10–20% Tris-Tricine gels (Bio-Rad) and autoradiography with Kodak film [21].

### ACTH Release from Permeabilized AtT-20 Cells

The permeabilization protocol used followed that described previously by others [13]. In brief, stably transfected or non-transfected AtT-20 cells were plated on tissue culture plates (5 × 10^4^/well) for 48 h, and subsequently rinsed with 0.1% BSA in DMEM, followed by permeabilization buffer (20 mM digitonin, 137 mM NaCl, 2.7 mM KCl, 5.6 mM glucose, 1 mg/ml BSA, 20 mM Hepes, pH 7.2), plus either 10 μM mastoparan (Neosystem Laboratoire, Strasbourg, France) or 100 μM GTPγS (Roche Molecular Biochemicals, Indianapolis, IN) at 37°C for 15 min [13,15,43]. To introduce antibodies into cells, affinity purified anti-calnuc (F-5059), anti-Gαi1/2 (AS), anti-Gαi3 (EC) or anti-GβIgG (30 μg/ml) were added and incubated with cells in permeabilization buffer at 37°C for 10 min according to a published protocol [44], followed by addition of 10 μM mastoparan for 15 min. Media were collected, and cells were lysed in 0.5% Triton X-100 at 4°C for 30 min, followed by centrifugation (14,000 × g for 5 min). ACTH in supernatants of both the medium and cell lysate were assessed by enzyme-linked immunosorbent assay (ELISA) using ACTH (Rat) EIAH kits (Peninsula Laboratories, San Carlos, CA) with a Vmax Kinetic Microplate Reader (λ = 450 nm) (Molecular Devices, Sunnyvale, CA). ACTH secretion was plotted as percent of total ACTH (secreted ACTH in supernatants *vs *secreted + intracellular ACTH). The results from each experiment were subjected to statistic analysis, and the final plotted results (mean ± SD) represent the average of values obtained in indicated separate experiments performed in either duplicate or triplicate as shown in figure legend.

### GTPγS Binding Assay

Purified recombinant His6-Gαi3 [45] and His6-calnuc [5] were prepared as described previously and GTPγS binding was assessed as described by others [23]. Mastoparan (100 μM) or His6-calnuc (1 μM) was incubated with 2 μM [^35^S]GTPγS (6000 cpm/pmol, NEN^® ^Life Science Products) [42] in the reaction buffer containing 10% glycerol, 1 mM DTT, 1 mM EDTA, 0.1 mM MgCl_2_, and 50 mM Hepes, pH 8.0. Reactions were started by addition of 200 nM His6-Gαi3 and incubated at 30°C for 5–60 min. At each time point, 50 μl of the reaction mixture were collected, diluted with ice-cold dilution buffer (160 mM NaCl, 0.2 mM GTP, 1 mM EDTA, and 20 mM Hepes, pH 8.0). Samples were collected on nitrocellulose filters by vacuum filtration. Filters were washed with ice cold washing buffer (25 mM MgCl_2_, 100 mM NaCl, and 25 mM Tris, pH 8.0). Bound radioactivity was determined by liquid scintillation counting. Total bound radioactivity never exceeded 2% of the total radioactivity.

### Subcellular Fractionation

Membrane (100,000 g pellets) and cytosolic fractions (100,000 g supernatants) were prepared by centrifugation of postnuclear supernatants from AtT-20 cells and analyzed by immunoblotting and ECL [3].

Light membranes containing PM and Golgi membranes were separated from heavy fractions containing ER and granules by centrifugation on discontinuous sucrose gradients using protocols similar to those previously published with minor modifications [3,5]. Briefly, postnuclear supernatants (PNS) prepared from AtT-20 cells were loaded on the top of sucrose step gradient containing 0.2, 0.4, 0.6, 1.0, 1.4, and 1.8 M in 1 mM Tris-HCl, pH 7.5, and centrifuged at 55,000 × g (SW60Ti rotor) for 2 h at 4°C. After centrifugation, 12 fractions were collected from the bottom, followed by centrifugation at 100,000 × g for 1 h. The resultant pellets were solubilized in Laemmli sample buffer and the solubilized proteins were separated on 10% Tris-Glycine or 10–20% Tris-Tricine gels (Bio-Rad) and analyzed by immunoblotting.

To separate PM from Golgi membranes sucrose gradient flotation [26] was applied as previously described [5] with minor modifications. In brief, postnuclear supernatants from AtT-20 cells were resuspended in 1.0 ml 1.3 M sucrose, followed by overlay with 0.5 ml of 1.2 M, 1.5 ml of 1.14 M, 0.5 ml of 0.99 M and 0.9 M sucrose, followed by centrifugation at 170,000 × g (SW60Ti rotor) for 15 h at 4°C. Eight fractions were collected by centrifugation (100,000 × g for 1 h), and membrane pellets were analyzed by SDS-PAGE followed by immunoblotting. Quantification of each band was performed by densitometry using Scan Analysis software (Biosoft, Cambridge, UK). Fraction density was determined using a digital refractometer (ABBE Mark II) (Cambridge Instruments, Buffalo, NY) as described [26].

## Abbreviations

APP: Alzheimer's β-amyloid precursor protein; RSG: regulated secretion granules; GAG: glycosaminoglycans; PM: plasma membrane; PNS: post nucleus supernatants; SS: signal sequence; ΔSS: deleting signal sequence; POMC: proopiomelanocortin; ACTH: adrenocorticotropic hormone; gACTH: glycosylated adrenocorticotropic hormone; GFP: green fluorescent protein; CFP: cyan fluorescent protein; CNG: calnuc-GFP; NT: non-transfected.

## Competing interests

The authors declare that they have no competing interests.

## Authors' contributions

PL designed, performed, analyzed data of majority of experiments, and wrote the manuscript. TF designed and performed many studies including GTPγS binding assay to investigate calnuc's biological function on regulating G protein activity. CL contributed Fig. 5. HH provided assistance to immunofluorescence, antibody affinity purification and many other related studies. MGF greatly supported with every aspect to the study and manuscript writing.
